# Autophagy-mediated regulation of neutrophil inflammatory responses and its relevance to central nervous system diseases

**DOI:** 10.3389/fnagi.2025.1702993

**Published:** 2025-12-17

**Authors:** Yuliu Li, Yiqing Tan, Wei Zuo

**Affiliations:** 1 Department of Pharmacy, Peking Union Medical College Hospital, Chinese Academy of Medical Sciences and Peking Union Medical College, Beijing, China; 2 State Key Laboratory of Bioactive Substance and Function of Natural Medicines, Institute of Materia Medica, Peking Union Medical College, Chinese Academy of Medical Sciences, Beijing, China

**Keywords:** autophagy, neutrophil, inflammation, central nervous system diseases, NETs

## Abstract

Autophagy is an intracellular degradation system, which plays a crucial role in regulating the inflammatory functions of neutrophils. Neutrophils, as crucial immunological phagocytes, are integral to inflammatory responses. In central nervous system diseases, neutrophils’ malfunction is closely associated with disease progression. Autophagy in neutrophils is highly conserved and plays a crucial regulatory role in both the biological functions and pathophysiological processes of neutrophils. In this review, we comprehensively explore the mechanisms of autophagy and its regulatory roles in various aspects of neutrophil biology, including the neutrophil life cycle, extracellular net traps (NETs) formation, degranulation, migration and adhesion, and phagocytosis. We also analyze the role of neutrophil autophagy in different central nervous system diseases such as Alzheimer’s disease, stroke, and neuroglioma. Regulating autophagy to control neutrophil inflammatory functions may emerge as a novel therapeutic strategy for treating central nervous system disorders.

## Graphical Abstract

**Figure:**
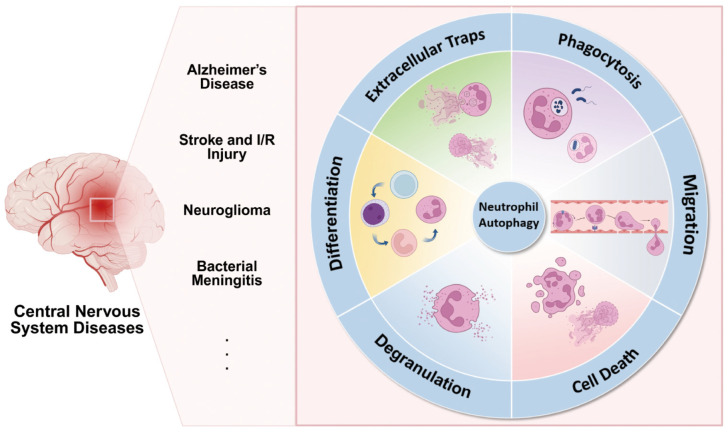
Autophagy orchestrates the terminal differentiation, survival, and key inflammatory effector functions of neutrophils—including neutrophil extracellular traps (NETs) formation, degranulation, chemotaxis/adhesion, and phagocytosis. The neutrophil inflammatory function related to autophagy plays a role in central nervous system diseases such as Alzheimer’s disease, stroke, glioma and bacterial meningitis. Drugs targeting neutrophil autophagy in the central nervous system have the potential for development and clinical application.

## 1 Introduction

Inflammation influences the disease process in central nervous system (CNS) injuries and diseases such as Alzheimer’s disease, stroke, and neuroglioma (Chitnis and Weiner, 2017). Central nervous system diseases are often characterized by the infiltration of inflammatory cells, including macrophages, microglia and astrocytes. Pro-inflammatory mediators amplify an inflammatory cascade, resulting in apoptotic, autophagic and degenerative changes in neurons and other neural cells (Chitnis and Weiner, 2017). Neutrophils are rapidly recruited to the site of inflammation and influence the inflammatory process through mechanisms such as degranulation, release of extracellular reticulocyte traps, and phagocytosis, which have important roles in the onset and progression of neurological diseases. However, most previous studies have focused on microglia in the central nervous system and macrophages in the peripheral nervous system (PNS; Balog et al., 2023), with little attention paid to the role of neutrophils. Additionally,

existing neutrophil-targeted therapies have shown limited clinical translation (Chen et al., 2025). This suggests that further research is needed to investigate the role of neutrophils in CNS diseases and potential therapeutic targets.

The proportion of neutrophils in all circulating leukocytes in the body is typically around 50%–70% under steady-state conditions (Mestas and Hughes, 2004), and these cells are functionally complex effector cells of the innate immune response, capable of regulating many pathophysiologic processes *in vivo*. The function of neutrophils is particularly important and complex during inflammation, and the neutrophil-lymphocyte ratios (NLR) is often used as a clinical prognostic indicator (Buonacera et al., 2022). NLR is closely related to inflammatory diseases and has been proven to be a reliable indicator for diagnosing bacteremia and sepsis (Gürol et al., 2015). In patients with glioblastoma, those with an NLR below 4.7 have a significantly longer progression-free survival period (Buonacera et al., 2022). In acute stroke, NLR may be a key factor in patient risk stratification (Li et al., 2021). On the one hand, neutrophils, as first responders to inflammation, are recruited to the site of inflammation, phagocytose tissue debris, and promote inflammation to subside; on the other hand, neutrophils also exacerbate inflammatory injury by releasing inflammatory factors and producing reactive oxygen species (Herrero-Cervera et al., 2022). In CNS disorders, neutrophils appear to be more active, potentially driving a vicious cycle that further increases the low-level chronic vascular inflammation associated with CNS disorders (Chakraborty et al., 2023).

Autophagy, an evolutionarily conserved intracellular degradation system, is a key mechanism involved in all aspects of neutrophil biology and pathophysiology. Autophagy plays an important role in maintaining cellular homeostasis in response to cellular stress by generating autophagosomes that envelop damaged organelles and misfolded proteins and transport them to lysosomes for degradation and recycling (Skendros et al., 2018). Autophagy in mammalian cells can be categorized into three main types based on the mode of intracellular substrate translocation to the lysosome: macroautophagy, microautophagy, and chaperone-mediated autophagy (Nie et al., 2021). Macroautophagy, the most extensively studied form of autophagy and the predominant pathway in neutrophil autophagy (Skendros et al., 2018), is the central focus of this paper and will hereafter be referred to as autophagy. The influence of neutrophils on the inflammatory process in CNS diseases is closely related to autophagy (Shrestha et al., 2020). Therefore, exploring autophagy-mediated changes in neutrophil inflammatory function may offer a novel therapeutic strategy for CNS diseases.

## 2 Mechanisms of autophagy

Autophagy is a mechanism that regulates intracellular homeostasis and is required for cells to cope with stresses such as starvation, hypoxia, oxidative bursts, DNA damage and infection. Cellular autophagy is regulated by a variety of genes, and these autophagy-related genes are uniformly named ATG genes (Klionsky et al., 2003). The isolation membrane, also known as the phagophore, is thought to originate from the endoplasmic reticulum (ER) or from lipid bilayers contributed by the trans-Golgi network and endosomes (Levine et al., 2011; Klionsky et al., 2003). Studies have also suggested that mitochondrial, plasma, and nuclear membranes may serve as additional sources for autophagosome membrane formation (Levine et al., 2011). Signals such as nutrient starvation induce autophagy through inhibition of mammalian target of rapamycin (mTOR) and activation of adenosine monophosphate-activated protein kinase (AMPK). This process leads to the translocation of mTOR substrate complexes, including unc-51-like autophagy-activating kinase 1/2 (ULK1/2), ATG13, focal adhesion kinase (FAK) family kinase-interacting protein of 200 kDa (FIP200), and ATG101, from the cytosol to certain domains of the endoplasmic reticulum or closely associated structures (Itakura and Mizushima, 2010; Mizushima, 2010). Autophagy is initiated when ULK1 undergoes dephosphorylation and dissociates from mammalian target of rapamycin complex 1 (mTORC1), accompanied by the phosphorylation of ATG13 and FIP200 (Gonzalez Porras et al., 2018; Gómez-Virgilio et al., 2022). The activated ULK complex (ULK1-ATG13-FIP200) targets the phosphoinositide 3-kinase (PI3K) complex, which is made up of beclin 1, vesicular protein sorting 15 (VPS15), VPS34, and ATG14. The ULK complex promotes local production of autophagosome-specific phosphatidylinositol-3-phosphate (PI3P; Kaur and Debnath, 2015). PI3P is essential for the elongation of the phagocytic vesicle. It is also essential for the recruitment of other ATG proteins to the vesicle (Xie and Klionsky, 2007). The ATG12-ATG5 and phosphatidylethanolamine (PE)-light chain 3 (LC3, an ATG8 homologue) are two interacting ubiquitin-like conjugates that play important roles in the process of elongation and completion of enclosure of the isolation membrane (Fujita et al., 2008; Parzych and Klionsky, 2014). The ATG12-ATG5 conjugate becomes a dimeric complex with ATG16L1 (Fujita et al., 2008), localizes to the outer membrane and promotes the lipidation of LC3 with PE (Gonzalez Porras et al., 2018). ATG4 cuts pro-LC3 to form LC3-I. ATG7 and ATG3 process LC3-I, conjugate to PE and form LC3-II, which is necessary for phagocyte elongation (Gómez-Virgilio et al., 2022). Immunofluorescence visualization of LC3-II is able to reflect autophagosome flux (Pankiv et al., 2007). Cargo-loaded autophagosomes mature through fusion with lysosomes, where their contents are degraded into amino acids and other by-products. These degradation products are then exported back into the cytoplasm by lysosomal permeases and transport proteins, and subsequently reused for macromolecular synthesis and cellular metabolism (Mizushima, 2007; Glick et al., 2010). Inhibition of mTOR using specific inhibitors such as rapamycin, Torin1 and PP242 induces autophagy. Inducers such as alginate can initiate autophagy via an mTOR-independent pathway, but the mechanism is currently unclear (Gonzalez Porras et al., 2018) (Table 1).

**TABLE 1:** Mechanisms of action of key signaling molecules in neutrophil autophagy and corresponding small-molecule modulators targeting these molecules.

Autophagy-related signal	Pathway and mechanism in neutrophil autophagy	Classical small-molecule modulators	References
mTOR	The mammalian target of rapamycin (mTOR) kinase is the catalytic subunit of two functionally distinct complexes, mTORC1 and mTORC2. mTORC1 phosphorylates ATG13 and ULK1/2, inhibiting the ULK complex that initiates autophagy. Inhibiting the mTOR pathway can accelerate the NETs release rate of neutrophils in response to bacterial stimulation.	Rapamycin is a allosteric mTORC1 inhibitor mTOR kinase inhibitors (mTOR-KIs), such as torin 1, block the phosphorylation of all mTORC1 substrates and induce autophagy.	Thoreen et al., 2009; Kim and Guan, 2015; Itakura and McCarty, 2013; Perez-Alvarez et al., 2018
AMPK	AMPK can sense low cellular ATP levels. AMPK can trigger autophagy in a double-pronged mechanism of directly activating ULK1 and inhibiting the mTORC1 complex1. AMPK phosphorylation affects the killing effect of neutrophils by enhancing neutrophils release of ROS and inhibit apoptosis to enhance phagocytosis and inhibit hyphal elongation.	AMPK activator drugs such as AICAR and Metformin are currently mostly used in *in vitro* or animal experiments. Metformin can induce autophagy by simultaneously activating AMPK and inhibiting mTORC1.	Herzig and Shaw, 2018; Mihaylova and Shaw, 2011; Si et al., 2022; Ornatowski et al., 2020; Kim and Guan, 2015
ULK	The mammalian ULK kinase complex is the is the core of the classical autophagy initiation It conveys a variety of autophagy-inducing signals to the downstream autophagic machinery, orchestrating the autophagic process from autophagosome initiation to their fusion with lysosomes.	The small molecule drugs such as SBI-0206965 and MRT68921 are all currently in the preclinical stage.	Mercer and Tooze, 2021; Egan et al., 2015; Chen et al., 2020
PI3K	In neutrophils, Class III PI3K (VPS34) initiates the classical autophagy and LC3-related phagocytosis by synthesizing PI3P. However, the highly expressed Class I PI3Kγ/δ negatively regulates autophagy and participates in chemotaxis and inflammatory signaling, jointly determining the autophagic activity and immune effect of neutrophils.	3-Methyladenine inhibits PI3KC3 and can block early autophagy, as well as inhibit the formation of NETs and long-lived neutrophil Gϕ.	Xiong et al., 2024; Dyugovskaya et al., 2014; Ornatowski et al., 2020
LC3	LC3 is localized on the autophagosome membrane through lipidation and drives the formation and maturation of autophagosomes. LC3-dependent autophagy plays a crucial role in NETosis; it forms LAPosome through non-classical autophagy pathways, promoting the fusion of phagosomes and lysosomes, and functioning in the clearance of apoptotic cells and pathogens by neutrophils.	DC-LC3in is a small molecule drug in the experimental stage. It can covalently target LC3 and weaken the lipidation of LC3B to inhibit autophagy.	Heckmann et al., 2017; Fan et al., 2021

**FIGURE 1:**
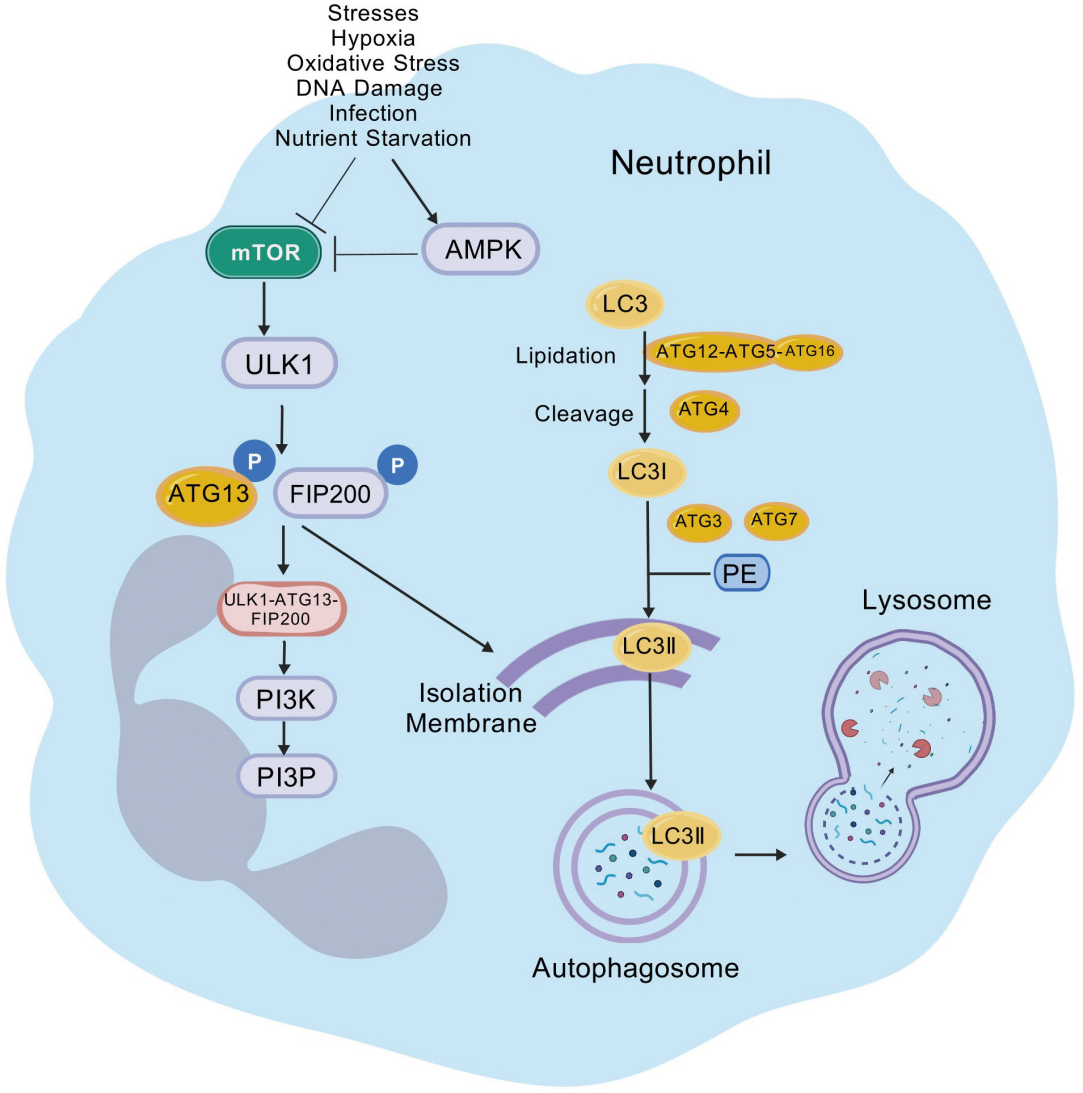
Regulation of autophagy in neutrophils. Autophagy in neutrophils can be initiated through multiple signaling pathways. In addition to common autophagy inducers such as nutrient starvation, neutrophils, as phagocytic cells, can also have autophagy induced by infection. With the combined action of various autophagy-related proteins, phagophores are generated from organelles such as the endoplasmic reticulum. These phagophores then expansion and close to become mature autophagosomes. After the autophagosomes fuse with lysosomes, the cargo is degraded and nutrients are recycled, completing the entire process of autophagy in neutrophils. mTOR, mammalian target of rapamycin; AMPK, adenosine monophosphate-activated protein kinase; ULK1, unc-51-like autophagy-activating kinase; FIP200, family kinase-interacting protein of 200 kDa; PI3K, phosphoinositide 3-kinase; PI3P, phosphatidylinositol-3-phosphate; LC3, light chain 3; PE, phosphatidylethanolamine.

In neutrophils, autophagy can be activated through multiple pathways, including both phagocytosis-dependent and phagocytosis-independent signaling (Itakura and McCarty, 2013; Mitroulis et al., 2010). These pathways promote autophagy by activating PI3K signaling, increasing reactive oxygen species (ROS) levels, and suppressing mTOR activity (Itakura and McCarty, 2013; Mitroulis et al., 2010; Huang et al., 2009; Goldmann and Medina, 2012). The G protein-coupled receptors (GPCRs) interacts with High mobility group box 1 (HMGB1) and Beclin-1 to initiate neutrophilic autophagy (Lv et al., 2017). Under nutrient starvation, neutrophils can initiate autophagy via the AMPK pathway (Chargui et al., 2012; Zhao and Klionsky, 2011), leading to the activation of ULK. After ULK and Beclin-1 mediate nucleation, autophagy proteins accumulate in phagosomes (Zhao and Klionsky, 2011; Ichimura et al., 2000; Parzych and Klionsky, 2014). In addition, a macrophage-induced Ca^2+^-dependent lectin receptor can initiate autophagy in neutrophils through activation of the Beclin-1 nucleation complex, in a manner that is independent of ROS and mTOR signaling (Sharma et al., 2017). The steps of membrane nucleation, cargo targeting, vesicle expansion, autophagosome formation, fusion with lysosomes, cargo degradation, and nutrient recycling occur after the initiation of autophagy, which ultimately completes the process of autophagy in neutrophils (Yu and Sun, 2020) (Figure 1).

## 3 Autophagy regulates neutrophil lifespan

### 3.1 Differentiation and generation

Neutrophils are derived from hematopoietic precursors in the bone marrow, with approximately 1 × 10^11^ neutrophils being born per second (Ng et al., 2019). When the bone marrow is stimulated by inflammatory cytokines, such as during an infection, emergent myelopoiesis occurs, and the production of neutrophils can be increased up to 1 × 10^12^ to replenish their numbers in the circulation (Zhang et al., 2025). Production of neutrophils is regulated in a strict cascade (Skendros et al., 2018), hematopoietic stem cells (HSC) differentiate into common myeloid progenitors (Liew and Kubes, 2019), myeloid progenitors undergo several stages of differentiation into neutrophils through myeloblasts (MBs), promyelocytes (MCs), metamyelocytes (MMs), and band cells (BCs; Nauseef and Borregaard, 2014; Riffelmacher et al., 2017). Autophagy plays an important role in neutrophil production and differentiation. Forkhead box O3 (FOXO3A)-mediated induction of autophagy has a protective effect on HSC, allowing them to survive metabolic stress. Autophagy controls fatty acid oxidation (FAO) and the mitochondrial respiratory chain pathway, providing sufficient ATP for energy-intensive differentiation processes. In the early stages of neutrophil differentiation, free fatty acids made by autophagy are important substrates for oxidative phosphorylation (OXPHOS). ATP deficiency during these stages slows down differentiation (Riffelmacher et al., 2017). The balance between hematopoietic stem cell maintenance and lineage differentiation is closely linked to energy metabolism (Suda et al., 2011; Mitroulis et al., 2018). In the highly hypoxic bone marrow microenvironment, HSC rely on glycolysis to meet their energy production needs (Suda et al., 2011; Takubo et al., 2013; Wang et al., 2014). Lipophagy, the autophagic ability to degrade fatty acid-enriched lipid droplets, provides free fatty acids for oxidative phosphorylation to enhance ATP production required for differentiation, leading to the conversion of glycolysis to oxidative phosphorylation (Riffelmacher et al., 2017). ATG12 deficiency results in the disruption of autophagy leading to the HSC’s metabolic reprogramming toward oxidative phosphorylation and myeloid lineage bias, producing a phenotype similar to that of activated HSC (Ho et al., 2017). ATG5 or ATG7 deficient neutrophil precursors exhibit an impaired lipophagy, mitochondrial respiration, and ATP production, which is accompanied by an increase in glycolytic activity leading to the differentiation of defective neutrophil precursors into metamyelocyte. Administration of free fatty acids can restore normal glucose metabolism in the neutrophil precursors with autophagy deficiency and promote their differentiation (Riffelmacher et al., 2017). In addition, several other factors are involved in autophagy regulation, such as the expression of ATG3, ATG4D, ATG5, and WIPI1 affects neutrophil differentiation by influencing the Ets-family hematopoietic transcription factor PU.1, which regulates autophagy through the microtubule-associated protein 1S (MAP1S, also known as C19ORF5) (Humbert et al., 2012; Rožman et al., 2015). Damage-regulator autophagy modulator 1 (DRAM-1) involved in all-trans retinoic acid (ATRA) -induced neutrophil differentiation (Humbert et al., 2012).

**FIGURE 2:**
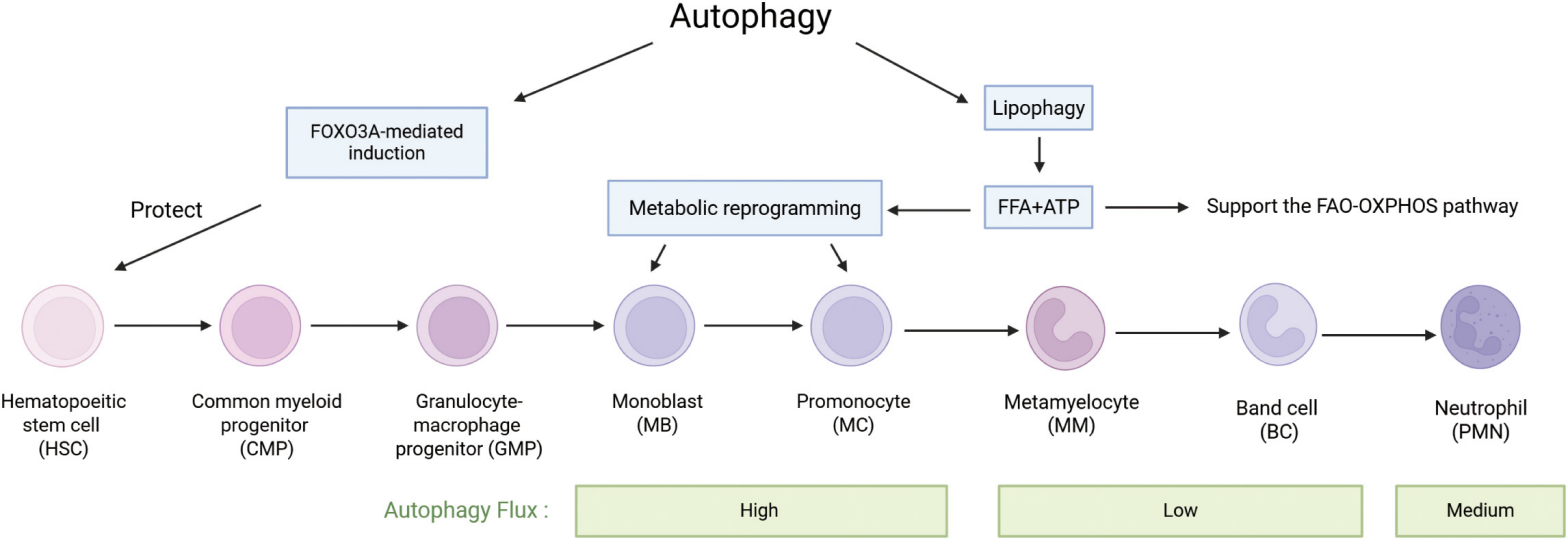
The role of autophagy in the differentiation of haematopoietic stem cells into neutrophils. HSCs undergo a series of stages to differentiate into neutrophils. During this process, autophagy induced by FOXO3A has a protective effect on HSCs. Free fatty acids (FFA) and ATP produced by lipophagy play important roles throughout the differentiation process, especially in MBs and MCs, which undergo significant metabolic reprogramming and a shift from glycolysis to fatty acid oxidation. MBs and MCs also exhibit the highest autophagy flux, which then decreases and subsequently increases again after differentiation into neutrophils.

The autophagy marker LC3-II flux was found to be significantly increased at the MB and MC stages, decreased at the MM and BC stages, and then slightly increased in mature neutrophils (Riffelmacher et al., 2017). Autophagic activity increases in myeloblasts, promyelocytes, and myleocytes, declines when differentiated into metamyleocytes and band cells, and is lowest in cells undergoing terminal differentiation (Riffelmacher et al., 2017; Rožman et al., 2015). A study reported differential expression of 22 autophagy-related genes in the differentiation of monocytes and granulocytes, suggesting that these genes may be important regulators involved in controlling the differentiation of granulocyte and monocyte progenitors (Huang et al., 2018). Overall, autophagy was up-regulated at the myeloid stage during neutrophil generation and differentiation, and progressively down-regulated as they continued to differentiate. These results suggest a link between autophagy and early neutrophil differentiation (Shrestha et al., 2020) (Figure 2).

### 3.2 Survival and death

Neutrophils have a short circulating half-life in the body (Pillay et al., 2010), but during inflammation, the lifespan of neutrophils significantly extends several times due to their activation (Colotta et al., 1992; Summers et al., 2010). This makes sure that neutrophils stay at the site of the infection and help reduce inflammation and repair tissue damage. Autophagy has a dual role in neutrophil fate. On the one hand, autophagy can promote cell survival by sensing oxidative stress and degrading damaged cellular components (Dyugovskaya et al., 2016). Autophagy plays a major role in regulating the production of neutrophil long-lived subpopulation giant phagocytes (Gφ). Gφ is larger in size than normal neutrophils and also survives for a longer time in cultures. Gφ is positive for CD15, CD66b, CD63, CD11b, myeloperoxidase (MPO), and neutrophil elastase (NE). It exhibits obvious autophagic features. For example, it has aggregation of light chain 3 beta (LC3B) and formation of LC3B-coated vacuoles (Lavie et al., 2017). Addition of the specific autophagy inhibitor 3-methyladenine (PI3K inhibitor) after 48 h of culture completely blocked Gϕ formation, and addition of bafilomycin A1 (which inhibits lysosomal acidification) at the early stage of culture (0 h) partially inhibited Gϕ formation; and addition after 48 h completely blocked Gϕ formation, suggesting that autophagy is critical in both early and maturation stages of neutrophil longevity isoforms of Gϕ production (Dyugovskaya et al., 2014).

Besides its role in promoting neutrophil survival, including the promotion of long-lived neutrophil subtypes, autophagy’s impact on neutrophil longevity is evident in the accelerating of neutrophil death. For example, autophagic-like cell death is a non-apoptotic form of cell death in neutrophils and is characterized by cytoplasmic vacuolization, nuclear condensation, mitochondrial swelling and plasma membrane integrity (von Gunten et al., 2005). Neutrophil autophagy induces adhesion molecules to trigger caspase-independent cell death. During this process, large vacuoles appear in the cytoplasm, resulting from the fusion of multiple cellular structures, including endosomes, autophagosomes, and secondary granules (Mihalache et al., 2011). B-cell lymphoma 2 (BCL-2) binds to Beclin 1/Atg6 and inhibits Beclin 1-mediated autophagy and autophagic cell death (Horn et al., 2018). Autophagy and intracellular ROS levels determine the form of neutrophil death (von Gunten et al., 2005; Mihalache et al., 2011), lower levels of ROS may lead to apoptosis (Conus et al., 2008; Geering et al., 2011), and at high ROS levels, neutrophils undergo necrosis associated with autophagy. However, the actual type of death in each cell is not necessarily the same when neutrophil cell populations are activated by certain death triggers *in vitro* (Mihalache and Simon, 2012). Human intravenous immunoglobulin (IVIg) preparations containing natural anti-Siglec-9 autoantibodies bind Siglec-9 on neutrophils, leading to autophagic-like cell death upon cytokine granulocyte/macrophage colony-stimulating factor (GM-CSF) initiation (von Gunten et al., 2006). Neutrophil extracellular traps (NETs) are extracellular effectors produced by neutrophils. Autophagy-induced “NETosis” has been suggested as a pathway of neutrophil death, distinct from apoptosis or necrosis. It involves amplification of nuclear material, chromatin decondensation, and disintegration of the nuclear membrane, leading to mixing of nuclear and cytoplasmic components. Eventually, the plasma membrane ruptures, releasing extracellular net traps into the surrounding environment (Fuchs et al., 2007; Metzler et al., 2014). Chromatin decondensation and NETs formation require autophagy. Wortmannin or 3-methyladenine (PI3K III inhibitors) or ATG5 or ATG7 defects can reduce NETosis in neutrophils by inhibiting autophagy, converting NETosis to apoptosis in specific circumstances (Sha et al., 2016; Xu et al., 2017; Ma et al., 2016). Although NETs production and neutrophil death do not always occur at the same time, a close link between the two can be established (Desai et al., 2016).

**FIGURE 3:**
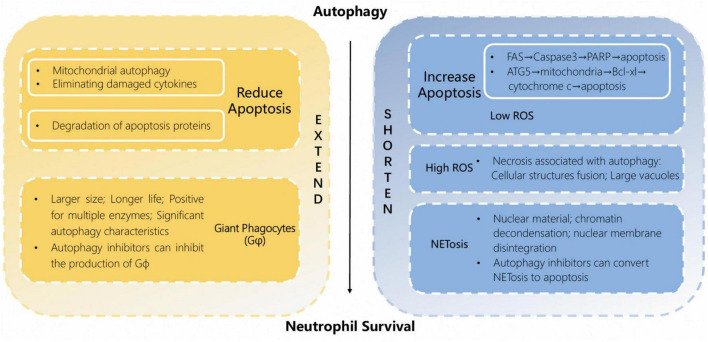
Autophagy has a dual effect on the lifespan and apoptosis of neutrophils. Autophagy can prevent neutrophil apoptosis through various mechanisms, including mitochondrial autophagy and the degradation of apoptosis-promoting proteins; there also exists an autophagy-mediated apoptosis pathway dependent on proteins such as ATG5 and caspase. Gφ is a neutrophil subpopulation associated with autophagy that exhibits a prolonged lifespan. NETosis is a programmed cell death pattern driven by the release of neutrophil extracellular traps (NETs) by neutrophils.

Autophagy also affects neutrophil survival and death by influencing apoptosis, and this role is bidirectional. On the one hand, autophagy can prevent neutrophils from undergoing intrinsic apoptosis through a variety of mechanisms, including mitochondrial autophagy and promotion of degradation of apoptotic proteins. Autophagy inhibits endoplasmic reticulum stress and helps neutrophils survive apoptotic stimuli (Hu et al., 2015). Reduced autophagy leads to a compensatory increase in apoptosis (Xu et al., 2017). Past experiments found that the use of autophagy inhibitors such as 3-methyladenine and chloroquine (CQ) significantly accelerated spontaneous apoptosis in neutrophils (Pliyev and Menshikov, 2012). On the other hand, there is significant autophagy-mediated apoptosis in neutrophils. In an *in vitro* model of immature neutrophils, cleaved Atg5 is transferred from the cytoplasm to the mitochondria, where it binds to the anti-apoptotic protein B-cell lymphoma-extra large (Bcl-XL), inducing the release of cytochrome c and thereby activating apoptosis (Xu et al., 2017; Huang et al., 2025). The apoptosis-protective agent BCL-2 can eliminate this pro-apoptotic function. Caspase protein family members are key regulators of apoptosis (Sahoo et al., 2023), cleavage of poly ADP-ribose polymerase (PARP) marks the onset of apoptosis (Fischer et al., 2003), autophagy activates both caspase 3 and PARP through the Fas-associated exogenous pathway. Caspase 8 can serve as a platform to activate autophagosomes (Xu et al., 2017). Autophagy can also induce apoptosis by degrading endogenous inhibitors such as the antiapoptotic proteins Cytochrome c oxidase subunit 6A1 (COX6A1), Myeloid cell leukemia-1 (Mcl-1; Xu et al., 2017). Overall, the pro-apoptotic activity of autophagy is relatively weaker than its anti-apoptotic activity, ultimately exerting an overall inhibitory effect on apoptosis (Yu and Sun, 2020) (Figure 3).

## 4 Autophagy regulation of neutrophil inflammatory function

### 4.1 Autophagy and NETs production

Neutrophil extracellular traps (NETs) are extracellular effectors produced by neutrophils and consisting of a dense reticulum of extracellular chromatin embedded with a variety of antimicrobial proteins (Urban et al., 2009), such as myeloperoxidase, neutrophil elastase, histones and others(Mitsios et al., 2016). NETs respond to a variety of stimuli, such as pathogens, exogenous compounds, inflammatory factors, platelets, and antibodies (Kenny et al., 2017; Remijsen et al., 2011; Peng et al., 2017; Maugeri et al., 2014). The cell death process that ends with the release of NETs is called NETosis and mediates pathogen capture and killing (Shrestha et al., 2020). In non-infectious diseases, NETs formation drives inflammatory responses, and neutrophils show heightened autophagic activity, neutrophils are characterized by increased autophagic activity (Chargui and El May, 2014). Although NETs can reduce inflammation by hydrolyzing cytokines and chemokines and help protect the host from pathogens, the presence of a non-specific proinflammatory component in NETs induces adjacent tissue damage by eliciting a proinflammatory response (Luo et al., 2023). In central nervous system diseases, NETs are involved in thrombosis, inflammation, blood-brain barrier disruption and neuronal damage (Luo et al., 2023). Inhibition of neutrophil extracellular trap formation ameliorates neuroinflammation and neuronal apoptosis in traumatic brain injury mice (Shi et al., 2023). The inflammatory environment also affects the nature of NETs released from neutrophils, e.g., Familial Mediterranean fever (FMF), a classic autoinflammatory disease, exhibits NETs carrying IL1β (Apostolidou et al., 2016).

**FIGURE 4:**
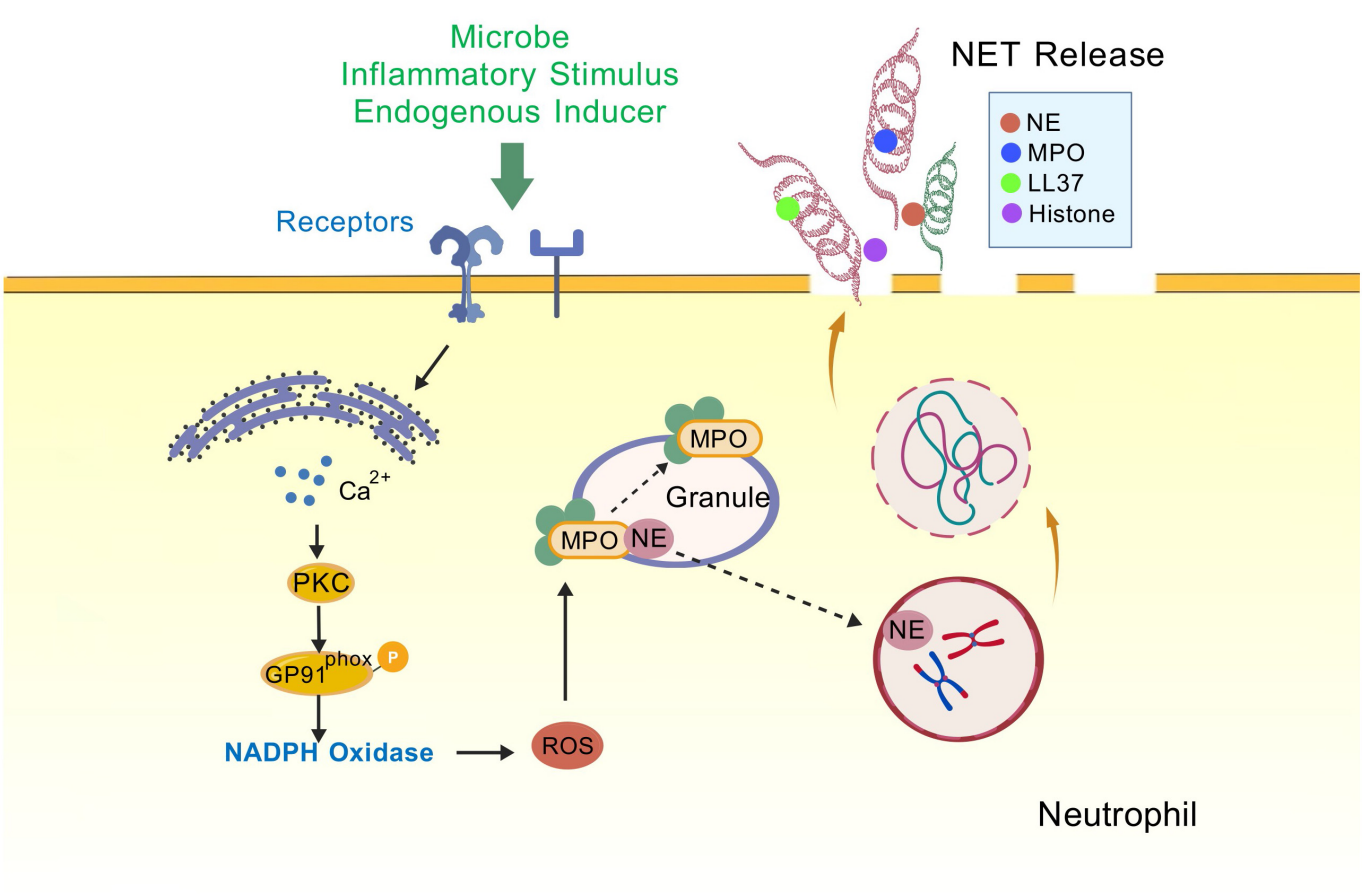
NETosis. Neutrophils are stimulated (e.g., by endogenous inducers, PMA, or LPS), triggering receptor binding on the cell membrane, opening membrane channels (not shown) that allow calcium from the endoplasmic reticulum to enter the cytoplasm and increase cytoplasmic calcium levels. The elevated calcium levels stimulate PKC activity, Gp91phox phosphorylation, and the assembly of functional NADPH oxidase, leading to the production of reactive oxygen species (ROS) and nitric oxide (NO) (not shown), which trigger the myeloperoxidase (MPO) pathway. In this pathway, MPO-mediated oxidative activation of neutrophil elastase (NE) is essential for NE to degrade the actin cytoskeleton in the cytoplasm and block phagocytosis. NE is then translocated to the nucleus, where it drives chromatin depolymerisation by processing histones. MPO also promotes chromatin depolymerisation. Morphological changes observed during NETosis include rupture of the nuclear membrane and granule membranes, as well as mixing of nuclear, granule, and cytoplasmic contents. Deimidation of histones and proteolytic cleavage may begin prior to nuclear disintegration and contribute to chromatin depolymerisation. Rupture of the plasma membrane allows the release of extracellular chromatin traps. PKC, protein kinase C; NADPH, nicotinamide adenine dinucleotide phosphate; GP91*^phox^* (NOX2), NADPH oxidase 2; MPO, myeloperoxidase; NE, Neutrophil elastase; LL37, Human Cathelicidin Antimicrobial Peptide.

The mechanism of NETs formation is not entirely comprehended; however, numerous studies have demonstrated that the process is closely associated with neutrophil autophagy. Autophagy positively regulates NETosis, and impaired autophagy is associated with reduced formation of NETs (Remijsen et al., 2011; Park et al., 2017; Sharma et al., 2017). The process of NETs formation involves a series of processes including ROS production, chromatin deconcentration, nuclear membrane disassembly, cell membrane rupture, and NETs release (Luo et al., 2023). In stages 1/4/5 of NETs formation, autophagy inhibits the respiratory burst, chromatin deconcentration, and induces histone citrullination (Mohammed et al., 2013; Itakura and McCarty, 2013; Iba et al., 2013); in stage 3, autophagy is involved in the externalization of membrane-bound proteins and cytoplasmic proteins (Xu et al., 2017; Kambas et al., 2012). Autophagy can induce NET generation in both ROS-dependent and ROS-independent ways (Remijsen et al., 2011). mTORC1, a protein complex downstream of the PI3K-Akt pathway, is one of the participants deregulated after ischemia and OGD, and is a key regulator of autophagy (Perez-Alvarez et al., 2018). Stimulation of neutrophils with bacterial-derived peptide followed by pharmacological blockade of the mTOR pathway inhibitors promotes neutrophil autophagy and accelerates the release of NETs (Itakura and McCarty, 2013). Neutrophils from patients with acute gouty arthritis exhibited autophagic activity and mediated NETs release, suggesting that autophagy-associated NETosis is associated with aseptic inflammation (Yang et al., 2019). Treatment of neutrophils with the autophagy inducer alginate significantly increased NETs formation (Guo et al., 2021). Both intracellular chromatin depolymerization and NET formation are indispensable for the occurrence of autophagy in phorbol myristate (PMA)-stimulated neutrophils (Remijsen et al., 2011) (Figure 4).

### 4.2 Autophagy regulates neutrophil degranulation

Autophagy regulates the initiation of neutrophil degranulation, which is one of the major mechanisms of the neutrophil inflammatory response (Bhattacharya et al., 2015), and excessive degranulation is a common feature of many inflammatory diseases. Neutrophils contain a diverse population of granules loaded with different proteins, including primary granules (azurophilic granules), secondary granules (specific granules), tertiary granules, and secretory vesicles (Cowland and Borregaard, 2016). The more toxic mediators, such as elastase, myeloperoxidase, defensins, histones, etc., are stored in primary granules (Borregaard and Cowland, 1997). Lactoferrin and other related proteins are stored in secondary granules, while matrix metalloproteinase-9 (MMP-9) and similar enzymes are stored in tertiary granules. Secretory vesicles contain proteins such as human serum albumin (Lacy, 2006). Autophagy is involved in the formation and release of neutrophil granules, and autophagy defects result in reduced degranulation of neutrophils *in vitro* and *in vivo*. Neutrophils from autophagy-deficient mice exhibit impaired ROS production, reduced secretion and release of the three major granule isoforms (MPO, lactoferrin, MMP-9), and reduced inflammatory function (Bhattacharya et al., 2015). In autophagy-deficient neutrophils, nicotinamide adenine dinucleotide phosphate (NADPH) oxidase-mediated generation of reactive oxygen species is also reduced, which suggests that NADPH oxidase is a player at the intersection of autophagy and degranulation, as its inhibition decreases neutrophil degranulation (Bhattacharya et al., 2015). Hypoxia stimulates PI3K signaling and reduces reactive oxygen species (ROS; Hartmann et al., 2008), both of which increase autophagy, and the expression of the autophagy marker protein LC3B-II in neutrophils increases (Lodge et al., 2020). Autophagy promotes degranulation by removing damaged mitochondria (mitochondrial autophagy) and oxidized proteins, thereby preventing the accumulation of intracellular ROS (Scherz-Shouval and Elazar, 2011). There is a reciprocal balancing effect between autophagy and ROS under hypoxia, and the potential impact of this effect on degranulation is complex and requires further elucidation (Lodge et al., 2020). It has been suggested that downregulation of the autophagy-related gene ATG7 is associated with reduced expression of the transcription factor CCAAT/enhancer-binding protein ε (C/EBPε), which is involved in myeloid differentiation and granule maturation, resulting in defective neutrophil granule maturation and reduced number and content of neutrophil granules (Riffelmacher et al., 2017). In this study, ATG7-deficient neutrophils demonstrated reduced levels of tertiary granule proteins while showing elevated levels of primary and secondary granule proteins. Since tertiary granules are formed at the band cell stage, and primary and secondary granules are continuously produced and degraded during neutrophil differentiation (Masson et al., 1969; Faurschou and Borregaard, 2003; Cowland and Borregaard, 2016; Gullberg et al., 1997), it is hypothesized that autophagy may be involved in the relevant transcriptional programs that regulate granule protein formation prior to the band cell stage (Shrestha et al., 2020).

### 4.3 Autophagy affects neutrophil migration and adhesion

Neutrophil recruitment involves multiple steps: adhesion, rolling, adhesion, crawling and transendothelial migration (Rodrigues et al., 2016). The migration of neutrophils to sites of inflammation is an important feature of the inflammatory process. Tissue-resident leukocytes encounter inflammatory mediators released by pathogens (such as cytokines, leukotrienes, and histamine), which cause changes on the surface of endothelial cells and initiate a neutrophil recruitment cascade. Chemokines induce directed crawling of neutrophils along chemokine gradients on the endothelium and accelerate their recruitment into target tissues (Massena et al., 2010). The adherence and migration of neutrophils facilitate their swift and effective exit from the vasculature into the tissues, a process in which adhesion molecules and integrins are crucial (Liew and Kubes, 2019). Autophagy influences chemokine production and release, controls neutrophil recruitment, and splenic tyrosine kinase (Syk) regulates neutrophil immune responses through kinase/rubicon-like autophagy-dependent pathways in mammals. Reduced production of chemokines, proinflammatory cytokines, neutrophil extracellular traps, reactive oxygen species, and myeloperoxidase blocked inhibition of neutrophil apoptosis and migration (Zhu et al., 2023). ATG5 autophagy-dependently controls the production of proinflammatory cytokines and chemokines during Mycobacterium tuberculosis infection, and reduces neutrophil recruitment (Kinsella et al., 2023). In late inflammation, recruited neutrophils die at the site of inflammation, and apoptotic neutrophils have been described to express “eat me” signaling and are subsequently engulfed by locally present phagocytes (Jeannin et al., 2008). Neutrophils can re-enter the circulation by “reverse migration” and return to the bone marrow for clearance, and defective neutrophil clearance is thought to contribute to the development of chronic inflammatory diseases (Dejas et al., 2023). The effect of autophagy on human neutrophil adhesion levels has not been well studied, but it has been found that in cows with fatty liver, neutrophil autophagy is enhanced, with four phases: early autophagic vacuoles, degradative autophagic vacuoles, glycogen vacuoles, and vacuoles, which produce adhesion defects (Peng et al., 2021). After histamine treatment of sub-acute rumen acidotic cows, co-localization between CD11b and LC3 was increased, suggesting that recirculation of adhesion molecules and autophagic fluxes were blocked, and neutrophil adhesion was increased (Wang et al., 2022). Furthermore, Genetic ablation of endothelial cell autophagy affects neutrophils. In various inflammatory models, it causes neutrophils to infiltrate tissues excessively and enhances their transendothelial migration (TEM) ability (Reglero-Real et al., 2021), playing an important role in neutrophil migration and adhesion.

### 4.4 Autophagy affects neutrophil phagocytosis

Autophagy and phagocytosis, two highly conserved clearance processes within neutrophils, share similar morphological features and functions, both of which are endogenous lysosome-dependent. Phagocytosis is central to the microbicidal function of neutrophils. Pathogens are phagocytosed into plasma membrane-derived phagosomes that mature with degradative properties (Lee et al., 2003). Neutrophil autophagy has an important impact on their phagocytosis. Several findings suggest that autophagy can regulate phagocytosis by affecting the expression of target-recognition receptors, phagosome maturation, and phagocytic receptors recycling (Lee et al., 2003), and influencing the efficiency of neutrophils in killing pathogens (Zhao and Klionsky, 2011). Multivesicular bodies formed by infected neutrophils through autophagy are able to fuse with pathogen-containing phagosomes (Griffiths and Mayorga, 2007). Sequential mobilization of neutrophil lysosomes or nuclear endosomes and the release of their cargo into autophagic vesicles are necessary events that mediate intracellular pathogen killing. In various microbial sepsis mouse models, after knocking out NLRP3 in peritoneal cells (mainly neutrophils), reduced autophagy, enhanced phagocytosis, and increased expression of scavenger receptors MARCO and mannose-binding lectin (MBL) were observed (Liliang et al., 2017). LC3-associated phagocytosis (LAP), which is often thought to be a crossover effect of autophagy and phagocytosis, can be treated as a novel non-classical autophagy or a specific type of phagocytosis (Galais et al., 2019). The characteristic of LAP is most evident in macrophages, but LC3 lipidation of phagosomes also exists in neutrophils. NOX2 and Rubicon are regarded as key proteins in the LAP process (Grijmans et al., 2022), although the latest research suggests that Rubicon is not essential in the LAP process (Gordon et al., 2022). The presence of LC3B in mouse and human neutrophil phagosomes requires activation of NADPH oxidase and ROS production (Huang et al., 2009; Mitroulis et al., 2010). During phagocytosis of bacterial pathogens or apoptotic and necrotic cells, TLR signaling and NADPH oxidase activation of LAP occur, leading to the attachment of LC3 to the cytoplasmic side of the phagosome membrane and facilitating phagosome maturation (Fletcher et al., 2018). Recruitment of LC3 to single-membrane phagosomes is dependent on the activity of autophagolytic enzymes Beclin-1, ATG5, and ATG7, and is independent of the recruitment of ULK1 (Sanjuan et al., 2007). Human neutrophils infected with Streptococcus pneumoniae *in vitro* are dependent on type III PI3K and ATG5 for autophagy, which enhances bacterial phagocytosis (Ullah et al., 2017). LC3-modified phagosomes called LAPosomes formed during this LAP process have a greater fusion capacity with lysosomes and enhanced degradation of the contained microorganisms compared to normal phagosomes (Herb et al., 2020). Integrin-mediated adhesion can initiate the engulfment action of neutrophils, and LAP further mediates it. Moreover, inhibiting the Vps34-UVRAG-RUBCN-containing PI3K complex has a blocking effect (Lu et al., 2024). LAP efficiently eliminates apoptotic neurons and abnormal protein aggregates, maintaining the homeostasis of the central nervous system. Thus, it plays a central role in the control of inflammation in various neurodegenerative diseases (Chen et al., 2024). Thus, autophagic cross phagocytosis protects the body from abnormal inflammatory responses.

Autophagy is capable of detecting and eliminating intracellular pathogens that escape from the endocytosis region of phagocytosis. Pattern recognition receptors (PRRs), including toll-like receptors (TLRs), nucleotide-binding oligomeric structural domain proteins (NOD)1/2, and ubiquitin-binding proteins p62/SQSTM1, are activated by the detection of diverse pathogen-associated molecular patterns (PAMPs). These patterns can trigger a specific form of autophagy at the cell membrane or within the cytoplasm, referred to as “xenophagy” (Deretic, 2011). Neutrophil phagocytosis prevents spillover of pro-inflammatory and neurotoxic molecules by ingesting extracellular material such as dying cells and pathogens. But excessive autophagy or dysfunctional phagocytosis can also exacerbate brain damage under certain pathological conditions (Galluzzi et al., 2016; Scheiblich and Bicker, 2017; Li et al., 2018). Thus, balancing the roles of autophagy and phagocytosis may be important for the treatment of certain neurological disorders.

## 5 The role of neutrophil autophagy in CNS diseases

Neutrophil inflammatory function exists to be clinically important in neurological disorders in which inflammation is prevalent, including Alzheimer’s disease (Thakur et al., 2023), stroke (Mohamud Yusuf et al., 2022), and gliomas (Kajiume and Kobayashi, 2018). Autophagy proteins play a role in the induction and suppression of neutrophil immune and inflammatory responses (Zhang et al., 2023). In neutrophil-mediated inflammation and autoimmune diseases, autophagy-deficient mice have reduced severity of including LPS-induced blood-brain barrier disruption (Bhattacharya et al., 2015). Thus, modulation of neutrophil inflammatory function through autophagy becomes a promising target, and pharmacological modulation of neutrophil autophagy may represent a novel strategy for the treatment of certain diseases (Table 2).

**TABLE 2:** The mechanism and clinical significance of neutrophil autophagy in the pathogenesis of neurological diseases.

Central nervous system diseases	Related mechanisms and therapeutic potential of neutrophil autophagy	References
Bacterial meningitis	NETs impair the host’s capacity for bacterial elimination; Phagocytosis and degranulation release antimicrobial proteins to clear invading pathogens	Mohanty et al., 2019; Nauseef and Borregaard, 2014
Multiple sclerosis	Inhibition of neutrophil NADPH oxidase activation reduces white matter injury in mice; MPO is an important component produced by neutrophil degranulation; inhibiting MPO reduces demyelination and axonal injury, and promotes oligodendrocyte regeneration and neurogenesis	Choi et al., 2015; Yu et al., 2018
Stroke / cerebral ischemia	MMPs produced by degranulation cause sustained damage in the early phase of ischemia and promote angiogenesis during recovery; After cerebral ischemia, the autophagy key protein mTOR is inhibited, promoting autophagy	Yang and Rosenberg, 2015; Kim et al., 2011
Alzheimer’s disease	Accumulation of NETs causes neuronal damage	Kretzschmar et al., 2021
Parkinson’s disease	CAP2 gene diagnoses Parkinson’s disease through NET-related immune activity Oxidative stress plays a key role in the onset and progression of PD	Li et al., 2024; Wang et al., 2025
Amyotrophic lateral sclerosis	The process of NET release causing peripheral motor pathway injury in ALS rats	Trias et al., 2018
Epilepsy	mTOR signaling pathway hyperactivation directly contributes to epilepsy	Sumadewi et al., 2023
Glioma	Glioma-derived IL-8 recruits infiltrating neutrophils to produce large amounts of NETs; Infiltrating neutrophils and myeloperoxidase-containing granules induce ferroptosis, promoting tumor necrosis in glioblastoma progression; Elastase secreted by infiltrating neutrophils also accelerates glioma infiltration	Zha et al., 2020; Yee et al., 2020; Iwatsuki et al., 2000

### 5.1 Alzheimer’s disease

The presence of chronic neuroinflammation, breaching of the blood-brain barrier (BBB), and increased levels of inflammatory mediators are central to the pathogenesis of Alzheimer’s disease (AD). Amyloid-beta (Aβ) is deposited in the brain, driving a persistent inflammatory process in AD patients. Previous studies have focused more on the role of microglia, the resident immune cells of the CNS, in AD, but recent evidence suggests that neutrophils infiltrate the cerebral vasculature and parenchyma and are involved in the regulation of immunity and inflammation (Zhang et al., 2024). Inflammatory responses in AD lead to hyperactivation of neutrophils, with marked changes in subsets of neutrophils (Sayed et al., 2020). Autophagy promotes the degranulation of neutrophils, and the released granular proteins have dual effects on neurons, both protective and toxic (Kasus-Jacobi et al., 2021). Inhibition of mtDNA-STING-NLRP3/IL-1β axis-mediated neutrophil infiltration and prevention of neutrophil migration into brain tissue protects neuronal health in the setting of Alzheimer’s disease (Xia et al., 2024). The aggregation of β-amyloid protein in neurons leads to the activation of the complement system, which induces the migration of neutrophils to the brain, triggering autophagy and subsequently the release of NETs (Kretzschmar et al., 2021). The accumulation of NETs and over-activation of the complement system leads to a cascade of inflammation that causes damage to neurons (Kretzschmar et al., 2021; de Bont et al., 2019). All of the above mentioned inflammatory functions of neutrophils are closely related to the occurrence of autophagy. The regulation of neutrophil degranulation, infiltration and migration, and NET generation through autophagy would be a potential therapeutic idea for Alzheimer’s disease. A NETs inhibitor, DNase, has been successfully applied in the treatment of Alzheimer’s disease. Genetic variants in the DNase gene, including DNASE1, DNASE2, and DNASE1L3, can lead to downregulation of DNase expression in Alzheimer’s disease (Kretzschmar et al., 2021; Tetz and Tetz, 2016).

### 5.2 Stroke and ischemia-reperfusion injury

After ischemic stroke, neutrophils are rapidly recruited into ischemic brain tissue and exacerbate stroke injury by releasing ROS, proteases, and proinflammatory cytokines (Mohamud Yusuf et al., 2022). Neutrophils may exacerbate ischemic microvascular injury due to cerebral capillary obstruction, leading to reperfusion defects during stroke recovery (Mohamud Yusuf et al., 2022). Cerebral ischemia-reperfusion diminishes ATP levels and activates the intracellular energy sensor AMPK, which subsequently inhibits mTORC1. This inhibition results in the dephosphorylation of the autophagy-related proteins Atg13 and ULK1, facilitating the formation of the ULK1 complex and ultimately expediting the initiation of autophagy (Yang et al., 2016; Huang et al., 2019; Zhang et al., 2022). mTORC1 is a protein complex downstream of the PI3K-Akt pathway involved in ischemic processes and post-OGD dysregulation (Perez-Alvarez et al., 2018). mTORC1 has multiple autophagy-inhibitory effects, including dissociating and inactivating the autophagy complex ULK promote and inactivating the phosphatidylinositol 3-kinase class III (PI3KCIII) complex at the initial and maturation stages of the autophagosome (Nakamura and Yoshimori, 2017). Following cerebral ischemia, insufficient energy supply leads to inhibition of mTORC1 activity. AMPK phosphorylates ULK1 at Ser317 and Ser777, triggering autophagy (Kim et al., 2011). Autophagy further promotes NETs generation and exacerbates cerebral reperfusion injury after ischemia. Mice with autophagy defects show reduced severity of neutrophil-mediated LPS-induced blood-brain barrier disruption (Bhattacharya et al., 2015). A bibliometric analysis study shows that the relationship between NETs and stroke is receiving increasing attention and has become a key research area (Xu et al., 2025). Autophagy ensures the complete formation and extracellular release of neutrophil granules by driving the maturation of granules and the ROS-NADPH oxidase signaling (Scherz-Shouval and Elazar, 2011). Degranulation produces MMP that initiates an injury cascade early in the acute hypoxic/ischemic phase, which persists over hours and days. But during injury and recovery from ischemic injury MMP degrades extracellular matrix (ECM) protein hydrolases, which have a protective effect on the cells and participate in regeneration of the damaged vasculature (Yang and Rosenberg, 2015).

### 5.3 Neuroglioma

Neutrophil autophagy plays a crucial role in the pathogenesis of CNS tumors, exhibiting both anti-tumor (N1) and pro-tumor (N2) phenotypes within the tumor microenvironment, and it possesses both pro- and anti-tumor functions (Coffelt et al., 2016). Neutrophils exhibit two phenotypes, N1 and N2, in cancer pathophysiology. Phenotypic polarization is frequently regulated by dynamic interactions within the tumor microenvironment, including cytokines, hypoxic conditions, and tumor cell signaling (Obeagu, 2025). Previous work has summarized the impact of autophagy on neutrophil differentiation, leading us to hypothesize that autophagy may also play a role in the N1-to-N2 and N2-to-N1 phenotypic transitions of neutrophils. For example, tumor microenvironment-induced autophagy may promote the survival and migration of pro-tumor (N2-like) neutrophils (Li et al., 2015). Autophagy-mediated neutrophil extracellular traps play a role in patients with malignant gliomas, and NETs produced by infiltrating neutrophils regulate the link between glioma and the tumor microenvironment through the mediation of the HMGB1/RAGE/IL-8 axis (Zha et al., 2020). And NET formation increases hypercoagulability in glioma patients (Zhang et al., 2021), suggesting that targeting autophagy-mediated neutrophilic extracellular traps may be an effective way to prevent thrombotic complications in glioma patients. Meanwhile, NETs impair the brain tumor barrier or brain-blood barrier, promoting the development and metastasis of gliomas (Lin et al., 2021). Neutrophil autophagy increases levels of the pro-metastatic proteins oncostatin M (OSM) and MMP-9, leading to tumor growth promoting cancer cell migration (Li et al., 2015). Impaired autophagy limits neutrophil degranulation and reduces the release of inflammatory molecules (Jin et al., 2018; Haimovici et al., 2014). LC3 is a key signal for neutrophil autophagy. The LAP mediated by LC3 plays a crucial role in promoting the transfer of neutrophil granules, triggering tumor cell death and necrosis expansion. Targeting this process is expected to improve the prognosis of glioblastoma (Lu et al., 2024). The core proteins of the LAP PI3KC3 complex are potential targets for developing novel cancer therapies (Lu and Li, 2025). Neutrophil autophagy also exhibits antitumor effects in some cases, such as the use of 5-fluorouracil (5-FU) to induce neutrophil autophagy, which eliminates neutrophils and improves survival in cancer patients (Kajiume and Kobayashi, 2018). Whether targeting neutrophil autophagy is beneficial in the treatment of neurological tumors deserves further investigation.

### 5.4 Bacterial meningitis

Bacterial meningitis is a prevalent and perilous type of meningitis resulting from bacterial infection of the soft meningeal arachnoid membranes and the cerebrospinal fluid within their enclosed cavities, as well as the fluid in the ventricles. The inflammatory response, caused by bacterial products, damages host cells and injured tissues used to isolate the causative agent (Nelson, 2006). During acute bacterial meningitis (ABM), large numbers of neutrophils are recruited to the CNS to cross the BBB to eliminate the bacteria (Tunkel et al., 2004). Neutrophil autophagy can mediate NET effects on the level of meningitis by influencing neutrophil phagocytosis and removing invading pathogens by promoting degranulation to release antimicrobial proteins/peptides (Nauseef and Borregaard, 2014). In a rat model of meningitis, disruption of NETs using DNase I significantly reduced bacterial load, suggesting that autophagy-mediated NETs reduce bacterial clearance and promote the development of pneumococcal meningitis *in vivo* (Mohanty et al., 2019). In addition to antimicrobial peptides, the degranulation products of neutrophils also contain substances that are toxic to cells. For example, MMP promotes granulocyte extravasation, acts as a convertase to promote the production and release of cytokines and chemokines, and impairs the blood-brain barrier, which is closely associated with the development of brain injury. The use of MMP inhibitors is also capable of inhibiting a variety of proteases such as factor α (TNF-α) converting enzyme (TACE), which is a key mediator of inflammation in bacterial meningitis. Combined inhibition of the protease and convertase activities of MMP has been found to protect the hippocampus from apoptotic injury and improve long-term neurological outcomes (Leib et al., 2001). The above study suggests that inflammatory functions such as neutrophil autophagy-mediated NET and degranulation may be involved in the development of bacterial meningitis, which is instructive for the control of inflammation and protection of the blood-brain barrier during the disease process.

## 6 Discussion

Under clinical conditions, multiple factors including oxidative stress, circadian rhythms, gender, psychological stress, and environmental influences play significant roles in regulating neutrophil autophagy. Under hypoxic conditions, ROS activated AMPK1, and then activated the autophagy protein ULK1 through phosphorylation at Ser317 and Ser777, or promoted autophagy by inhibiting downstream mTOR kinase activity (Kaminskyy and Zhivotovsky, 2014). The circadian rhythm affects the autophagy-related functions of neutrophils. In human neutrophils, granule density and protein content oscillate diurnally, peaking in degranulation during the afternoon; accordingly, fresh-like cells in the early morning display a superior NET-forming capacity compared with their aged-like counterparts in the afternoon (Adrover et al., 2020). Psychological stress primarily impairs neutophil autophagy-related inflammatory functions via neuroendocrine pathways. Under chronic stress, memory, cognition and behavior, as well as whole-body homeostasis—including the cardiovascular, digestive and immune systems—are all affected (Yaribeygi et al., 2017). Chronic stress disrupts the normal circadian rhythm of neutrophils and, through glucocorticoid release, increases NETs formation (He et al., 2024). Gender differences also influence neutrophil autophagy (Richter et al., 2025), potentially explaining sex-specific variations observed in various diseases and physiological states between males and females.

At present, small molecule inhibitors or agonists targeting the autophagy process are being extensively studied. The highly selective ULK1 kinase inhibitor SBI-0206965 can inhibit the phosphorylation events mediated by ULK1 within the cells, thereby regulating autophagy and cell survival (Egan et al., 2015). Spermidine acts as an autophagy enhancer, and the gene regulatory changes mediated by it in neurodegenerative diseases, including Alzheimer’s disease (AD) and Parkinson’s disease (PD), such as Beclin-1, LC3-II and p62, may affect the autophagy process in these neurodegenerative diseases (Satarker et al., 2024). Metformin activates the AMPK pathway to enhance cellular energy metabolism and autophagy, addressing oxidative stress issues in neurodegenerative diseases and neuroinflammation, and has neuroprotective capabilities for diseases such as Parkinson’s disease (Kruczkowska et al., 2025), Alzheimer’s disease, Huntington’s disease and multiple sclerosis. There are also small molecule substances that exert effects, such as dexmedetomidine, which inhibits excessive autophagy by upregulating HIF-1α (Zhu et al., 2021); Fingolimod activates the mTOR/p70S6K pathway, reduces autophagosomes and beclin 1, and inhibits autophagy (Li et al., 2017).

The role of neutrophil autophagy in inflammatory functions is not static and is closely associated with disease progression. First, autophagy within neutrophils is not a singular process. It is considered a double-edged sword in neutrophils: while it aids cell survival by detecting oxidative stress and clearing damaged cellular components, it can also harm cells and accelerate cell death (Yu and Sun, 2020). During disease progression, such as in cerebral ischemia, early autophagy activation maintains the intracellular environment by degrading misfolded proteins and damaged organelles, thereby exerting neuroprotective effects (Wang et al., 2013). However, sustained autophagy activation during reperfusion exerts detrimental effects on the brain (Pluta, 2023). In gliomas, neutrophil autophagy simultaneously follows two opposing pathways: “NETs-promoting metastasis/thrombosis” and “granzyme-promoting antitumor effects,” playing a dual role in both accelerating tumor progression and enhancing anticancer efficacy (Li et al., 2015).

Central nervous system (CNS) diseases remain a major global public health challenge, yet the drug-development success rate in this field is substantially lower than that observed in many other therapeutic areas (Morofuji and Nakagawa, 2020). Drug development success rates for disorders such as Alzheimer’s disease, stroke, and brain tumors remain particularly low, reflecting, at least in part, an insufficient understanding of their pathological mechanisms and limitations in current target-selection strategies (Morofuji and Nakagawa, 2020). In CNS disorders, circulating and brain-infiltrating neutrophils have been shown to contribute to disease progression and secondary tissue injury (Bui et al., 2022). Modulating neutrophil autophagy is therefore expected to attenuate acute neurological damage while also mitigating chronic inflammation–driven neurodegenerative processes. The feasibility of targeting autophagy has been demonstrated by existing small-molecule agents—including certain mTOR regulators and AMPK agonists—that are capable of altering autophagy flux *in vivo* (Rubinsztein et al., 2012). Consequently, therapeutics directed at neutrophil autophagy hold promise for addressing the current scarcity of effective CNS therapies and may facilitate more efficient clinical translation strategies.
